# *Microgloma* Sanders & Allen, 1973 (Nuculanidae) and *Pristigloma* Dall, 1900 (Pristiglomidae) (Pelecypoda) in the Campos Basin off Brazil

**DOI:** 10.3897/zookeys.152.1646

**Published:** 2011-12-08

**Authors:** Natalia Pereira Benaim, Ricardo Silva Absalão

**Affiliations:** 1 Universidade Federal do Rio de Janeiro, Departamento de Zoologia, Rua Professor Rodolpho Paulo Rocco, Ilha do Fundão, CEP 21941901

**Keywords:** Pristiglomidae, Nuculanidae, Pelecypoda, deep-sea, biodiversity, Campos Basin slope, Rio de Janeiro

## Abstract

As a secondary result of oil prospecting in Brazil, samples from the Campos Basin continental slope became available. In these samples, specimens of the genera *Microgloma* Sanders & Allen, 1973 and *Pristigloma* Dall, 1900 were found. This contribution provides the southernmost record of the genus *Microgloma*, the first record of *Microgloma mirmidina* (Dautzenberg & Fischer 1897) from the western Atlantic, the descriptions of *Microgloma macaron* **sp. n.** and *Microgloma nhanduti* **sp. n.**as new species, and the shallowest record of *Pristigloma alba* Sanders & Allen 1973.

## Introduction

Knowledge of the deep-sea mollusks from the Campos Basin has increased in the last ten years, and several new species have been described (Absalão et al. 2001, 2003; Absalão and Pimenta 2003, 2005; Caetano et al. 2006; Zelaya et al. 2006; Pimenta et al. 2008; Oliveira and Absalão 2007, 2008, 2009, 2010a, 2010b; Absalão 2009; Benaim and Absalão 2011). However, the genera *Microgloma* Sanders & Allen, 1973 and *Pristigloma* Dall, 1900 have not been recorded until the present report.

*Microgloma* and *Pristigloma* comprise a few species that appear represented only in the Atlantic Ocean, each genus being represented by no more than five species. Their systematic affinities have been the subject of debate for almost 30 years (Sanders and Allen 1973, Allen and Hannah 1986, Ockelmann and Warén 1998, La Perna 2003, 2008), reflecting the usual confusion in protobranch taxonomy. Both *Pristigloma* and *Microgloma* were considered as members of the Pristiglomidae by Sanders and Allen (1973) and subsequent authors, until Ockelmann and Warén (1998) revised the systematic affinities of the genus *Microgloma*. This genusis characterized by miniaturization, and was considered by Sanders and Allen (1973) to be among the smallest pelecypods known. Synapomorphies include the enlarged innermost teeth of the left valve and the radially wrinkled surface of the prodissoconch. Members of this genus may comprise progenetic representatives of the family Nuculanidae, and may represent a polyphyletic group (Ockelmann and Warén 1998, La Perna 2008).

The five species of *Microgloma* described until now [*Microgloma tumidula* (Monterossato, 1880), *Microgloma mirmidina* (Dautzenberg & Fischer, 1897), *Microgloma yongei* Sanders & Allen, 1973, *Microgloma pusilla* Jeffreys, 1979, and *Microgloma guilornadi* Hoeksema, 1993] were recorded from Western Europe (Iberian Peninsula, Mediterranean Sea, Azores and Canaries), West Africa (Cape Verde and Angola), Surinam, and North America. Prior to the report of Ockelmann and Warén (1998), there was no record of the genus *Microgloma* for the western Atlantic; these authors suggested the possibility of the presence of some specimens of *Microgloma* in Surinam, but made no formal record. Allen (2008) presented a checklist of the pelecypods of the Atlantic and made reference to the presence of *Microgloma pusilla*, *Microgloma tumidula* (as *Microgloma turnerae*), *Microgloma yongei*, and *Microgloma* sp. in North America and Surinam.

*Pristigloma* is a genus widespread in the entire Atlantic Ocean and is characterized by smooth, fragile shells, lamellar hinge teeth with an unequal number of teeth on both plates, and a large, internally elongated ligament which is opisthodetic (Sanders and Allen 1973).

Here, we present the first records of the genus *Microgloma* from the southwestern Atlantic, as well as descriptions of two new species belonging to this genus. We also present new points in the geographical distribution of *Pristigloma alba* Sanders & Allen 1973.

## Material and methods

The samples used in the present study were collected by means of a box corer in the Campos Basin, off Rio de Janeiro State (22°S, 41°W), Brazil, from the research vessel *Astro–Garoupa*, as part of the programs “Environmental Characterization of Campos Basin, RJ, Brazil" during the years 2002 and 2003, and “Habitats Project – Campos Basin Environmental Heterogeneity" in 2008 and 2009. Both programs were sponsored by the Brazilian oil company Petrobras S.A. Of the material obtained we observed 260 samples taken between the isobaths of 400 and 2500 m. The list of localities with *Microgloma* and/or *Pristigloma* specimens is given in Tables 1 and 2. Most of the shells were found in a good state of preservation, with valves attached, ligaments intact, and often with the mass of the animal body inside the shell. Unfortunately, there were no preserved organs in these cases. Each specimen was examined under stereoscopic microscope (Nikon SMZ 800), and selected specimens were photographed with a scanning electron microscope (ZEISS EVO 40), at the Gerência de Bioestratigraﬁa e Paleoecologia Aplicada (BPA), of the Petrobras Research Center (Centro de Pesquisas da Petrobras, CENPES).

**Table 1.** Table of the localities sampled as part of the project Environmental Characterization of Campos Basin.

**Station**	**Depth (m)**	**Latitude / Longitude**	**Date**
10	1700	21°58'36.06"S, 39°46'30.28"W	08/10/2001
28	1930	22°06'52.98"S, 39°44'13.90"W	08/05/2002
32	900	22°38'01.14"S, 40°17'26.55"W	18/05/2002
33	900	22°35'47.22"S, 40°15'00.33"W	18/05/2002
34	900	22°33'31.21"S, 40°12'05.38"W	18/05/2002
36	1000	22°37'54.17"S, 40°13'36.46"W	19/05/2002
37	1000	22°39'44.28"S, 40°15'44.41"W	19/05/2002
38	1100	22°41'18.79"S, 40°14'05.93"W	15/05/2002
42	1200	22°41'39.45"S, 40°10'24.84"W	15/05/2002
47	1650	22°11'04.40"S, 39°47'04.60"W	25/11/2002
48	1950	22°11'16.63"S, 39°43'44.70"W	25/11/2002
51	1350	22°04'43.44"S, 39°49'08.29"W	24/11/2002
52	1650	22°04'44.26"S, 39°46'31.55"W	24/11/2002
53	1950	22°04'46.20"S, 39°43'02.02"W	24/11/2002
54	750	21°57'17.50"S, 39°56'01.10"W	12/12/2002
57	1650	21°57'15.55"S, 39°47'43.80"W	14/12/2002
58	1950	21°57'26.87"S, 39°40'33.80"W	11/12/2002
59	750	21°52'59.60"S, 39°55'30.60"W	12/12/2002
61	1350	21°52'51.90"S, 39°48'11.68"W	12/12/2002
62	1650	21°52'41.91"S, 39°46'17.52"W	11/12/2002
63	1950	21°52'44.10"S, 39°40'45.60"W	11/12/2002
64	750	22°36'03.00"S, 40°21'45.36"W	22/11/2002
65	1050	22°40'57.81"S, 40°16'30.35"W	22/11/2002
68	1950	22°48'05.28"S, 40°06'38.64"W	15/11/2002
69	750	22°31'12.47"S, 40°15'11.08"W	22/11/2002
73	1950	22°41'35.24"S, 40°00'45.24"W	22/11/2002
74	750	22°27'31.62"S, 40°09'23.19"W	21/11/2002
75	1050	22°31'28.28"S, 40°03'50.40"W	19/11/2002
77	1650	22°36'03.37"S, 39°57'54.68"W	16/11/2002
78	1950	22°37'02.47"S, 39°56'20.52"W	23/11/2002
81	1350	22°27'18.98"S, 39°54'50.48"W	17/11/2002
83	1950	22°30'35.35"S, 39°51'45.42"W	23/11/2002
85	1350	22°29'33.89"S, 39°56'17.64"W	19/11/2002
86	1650	22°31'36.00"S, 39°55'15.00"W	16/11/2002
87	1950	22°33'10.00"S, 39°54'22.00"W	23/11/2002
45	1050	22°10'53.40"S, 39°52'18.30"W	01/07/2003
46	1336	22°10'54.60"S, 39°48'59.50"W	25/06/2003
48	1968	22°11'16.50"S, 39°43'44.60"W	22/06/2003
49	722	22°04'32.80"S, 39°54'11.40"W	30/06/2003
52	1643	22°04'45.20"S, 39°46'31.70"W	27/6/2003
53	1910	22°04'45.40"S, 39°41'58.50"W	27/6/2003
54	698	21°57'11.80"S, 39°56'04.20"W	29/06/2003
56	1357	21°57'15.60"S, 39°49'37.50"W	25/06/2003
58	1942	21°57'26.80"S, 39°40'34.00"W	27/06/2003
59	750	21°52'59.20"S, 39°55'32.20"W	29/06/2003
61	1350	21°52'51.80"S, 39°48'12.50"W	26/06/2003
63	1941	21°52'43.10"S, 39°40'41.60"W	26/06/2003
64	750	22°36'01.30"S, 40°21'43.70"W	11/06/2003
65	1050	22°40'57.70"S, 40°16'31.10"W	11/06/2003
67	1596	22°46'58.30"S, 40°07'49.30"W	12/06/2003
68	1972	22°48'05.90"S, 40°06'38.60"W	12/06/2003
69	743	22°31'11.80"S, 40°15'12.10"W	18/06/2003
71	1350	22°38'52.90"S, 40°04'16.30"W	14/06/2003
75	1050	22°31'28.30"S, 40°03'49.30"W	18/06/2003
77	1650	22°36'12.20"S, 39°58'22.90"W	13/06/2003
78	1945	22°37'02.90"S, 39°56'20.10"W	13/06/2003
82	1650	22°28'46.50"S, 39°53'27.90"W	17/06/2003
84	1050	22°26'28.80"S, 39°58'53.30"W	20/06/2003
86	1630	22°31'37.20"S, 39°55'14.50"W	16/06/2003
87	1934	22°33'08.00"S, 39°54'21.50"W	15/06/2003

**Table 2.** Table of the localities sampled as part of the Habitats Project – Campos Basin Environmental Heterogeneity.

**Cruise**	**Station (#)**		**Depth (m)**	**Latitude / Longitude**	**Date**
HAB 4	D11	R1	2449	22°52'15.30"S, 40°05'10.40"W	22/5/2008
HAB 4	G12	R1	3236	22°12'19.50"S, 38°35'52.00"W	25/5/2008
HAB 6	D07	R1	698	22°36'27.10"S, 40°22'29.60"W	25/6/2008
HAB 6	D07	R2	700	22°36'27.30"S, 40°22'29.30"W	25/6/2008
HAB 6	D07	R2	700	22°36'27.30"S, 40°22'29.30"W	25/6/2008
HAB 6	A7	R1	694	23°39'20.10"S, 41°18'30.30"W	23/6/2008
HAB 6	A7	R2	692	23°39'19.80"S, 41°18'30.20"W	23/6/2008
HAB 6	A7	R2	692	23°39'19.80"S, 41°18'30.20"W	23/6/2008
HAB 6	A7	R3	733	23°39'19.90"S, 41°18'30.50"W	24/6/2008
HAB 6	CANAC7	R1	758	21°47'26.70"S, 40°02'13.30"W	28/6/2008
HAB 6	CANAC7	R2	753	21°47'26.60"S, 40°02'13.70"W	28/6/2008
HAB 6	I07	R1	694	21°11'12.20"S, 40°12'52.00"W	29/6/2008
HAB 6	D07	R2	700	22°36'27.30"S, 40°22'29.00"W	25/6/2008
HAB 6	D07	R2	700	22°36'27.30"S, 40°22'29.30"W	25/6/2008
HAB 6	D07	R1	698	22°36'27.10"S, 40°22'29.60"W	25/6/2008
HAB 6	D07	R2	700	22°36'27.30"S, 40°22'29.30"W	25/6/2008
HAB 7	D06	R1	396	22°33'35.70"S, 40°26'38.90"W	08/7/2008
HAB 7	D06	R3	393	22°33'33.80"S, 40°26'40.30"W	11/7/2008
HAB 7	H7	R1	700	21°41'12.30"S, 40°02'20.20"W	07/7/2008
HAB 7	H7	R2	699	21°41'11.70"S, 40°02'20.70"W	07/7/2008
HAB 7	H7	R3	700	21°41'11.80"S, 40°02'20.40"W	07/7/2008
HAB 7	I07	R3	792	21°11'02.60"S, 40°12'18.20"W	05/7/2008
HAB 8	D06	R2	401	22°33'35.10"S, 40°26'37.50"W	31/1/2009
HAB 8	D07	R2	696	22°36'25.30"S, 40°22'30.60"W	29/1/2009
HAB 8	C10	R3	1953	23°08'23.80"S, 40°36'37.90"W	27/1/2009
HAB 8	A07	R2	701	23°39'20.60"S, 41°18'28.20"W	28/1/2009
HAB 8	A07	R3	693	23°39'21.90"S, 41°18'33.10"W	28/1/2009
HAB 9	CANAC7	R2	780	21°47'26.60"S, 40°01'55.30"W	06/2/2009
HAB 9	CANAC7	R3	775	21°47'26.70"S, 40°01'55.50"W	06/2/2009
HAB 9	CANG–7	R2	720	21°56'11.90"S, 39°57'45.30"W	07/2/2009
HAB 9	H07	R2	702	21°41'12.60"S, 40°01'56.10"W	06/2/2009

**Figures 1–2.**
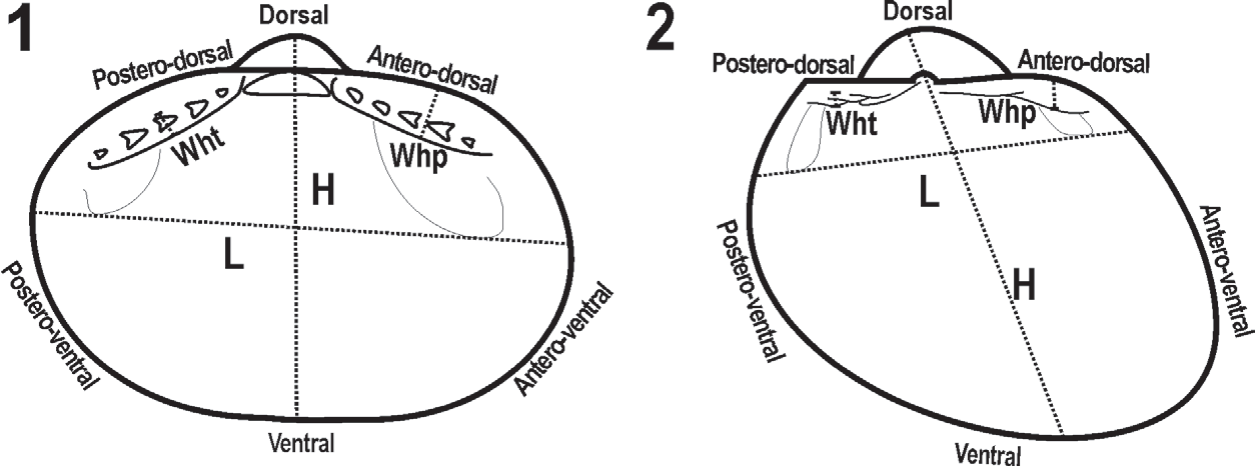
Scheme of the measurements and abbreviations used in the descriptions of the species **A** *Microgloma* **B** *Pristigloma* **H** height **L** length, wht – width of hinge teeth, whp – width of hinge plate.

Taxonomic identifications were made through comparison with the figures of type specimens [*Microgloma pusilla* (Jeffreys, 1879)] and descriptions available in the literature (Sanders and Allen 1973, Salas 1996, Ockelmann and Warén 1998, La Perna 2008, Oliver et al. 2009). The species were characterized considering traditional criteria used in pelecypod orientation and terminology (Figs 1–2) (Fischer 1886, Sanders and Allen 1973, Mikkelsen and Bieler 2008, Baylei 2009). In view of the importance of the features of the hinge plate for the discrimination of other protobranch species (Benaim and Absalão 2011, Benaim et al. 2011), and also some subjective concepts in taxonomy (e.g., ‘thin’ or ‘thick’), we described the species using certain quantitative criteria such as the ratios of the hinge teeth (wht) and hinge plate (whp) measurements (Figs 1–2), which are described as follows: ‘thin’ for width of hinge plate/total height ratio < 0.1; ‘thick’ for width of hinge plate/total height ratio ≥ 0.1. The width of the hinge teeth was measured just above (dorsal) and below (ventral) the limit of the bigger teeth. The width of the hinge plate was measured in the thicker part with a straight line (Figs 1–2).

The material analyzed in this study is deposited in the Mollusca collections of the following institutions: Departamento de Zoologia, Instituto de Biologia, Universidade Federal do Rio de Janeiro (IBUFRJ); Museu Nacional do Rio de Janeiro (MNRJ); Museu de Zoologia da Universidade de São Paulo (MZUSP); Museu Oceanográfico da Fundação Universitária de Rio Grande (MOFURG); National Museum of Natural History, Smithsonian Institute (USNM); and Muséum National d’Histoire Naturelle, Paris (MNHN). The following abbreviations are used: # – station; IBUFRJ – Instituto de Biologia da Universidade Federal do Rio de Janeiro; MNRJ – Museu Nacional do Rio de Janeiro; MZUSP – Museu de Zoologia da Universidade de São Paulo; MOFURG – Museu Oceanográfico da Fundação Universitária de Rio Grande; MNHN – Muséum National d’Histoire Naturelle, Paris; USNM – National Museum of Natural History, Smithsonian Institute; MCZ – Museum of Comparative Zoology, Harvard University, Cambridge, U.S.A.

## Systematics

### Pristigloma alba

Sanders & Allen, 1973

http://species-id.net/wiki/Pristigloma_alba

Figs 3–8

Pristigloma alba Sanders & Allen, 1973: 245, fig 5; Allen 2008: 67, 87, 95, 97–101, 103, 111, 113, 119, 141, 146, 152, 153, 157, 167, 168, 173. Oliver et al. 2009: figs. MO11691-11697 (available online).

#### Type specimen. 

MCZ 271976. We tried to find this lot in the MCZ with the help of Mr. Cleo Oliveira, but the curators could not find it. We made contact with Dr John A. Allen who sent us live specimens from Surinam Basin #293 (08°58'N54°04'W, 1518 m) to compare with Campos Basin specimens. Furthermore, using the good description in Sanders and Allen (1973) and figures of the specimens of Rockall Trough and Biscay Bay available in Oliver et al. (2009) we could properly identify our specimens.

**Figure 3–8.**
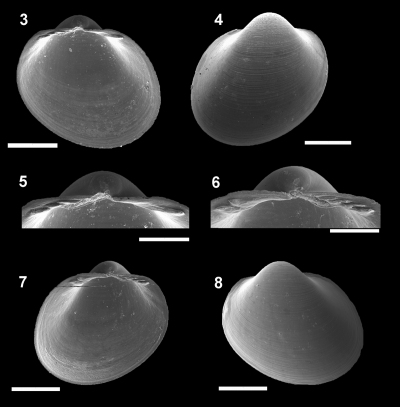
*Pristigloma alba* Sanders & Allen, 1973 IBUFRJ 16161. Left valve: internal view **3** external view **4** detail of the hinge plate **5** Right valve: detail of the hinge plate **6** internal view **7** external view **8** Scale bars 3–4, 7–8 = 500 μm, 5–6= 250 μm

#### Geographical distribution. 

Angola, 3739–4597 m; Canaries, 6709–6711 m; North America, 2178–4892 m; Brazil, 3459 m; Argentina, 4382–4405 m (all from Sanders and Allen 1973); Cape Verde, 3495 m; Angola, 3797 m; Canaries, 2351–3000 m; West Europe, 2897–4660 m; Newfoundland, 4400 m; North America, 2178–4833 m; Surinam, 5100 m; Brazil, 3495 m; Argentine, 4402 m (all from Allen 2008); Rockall Trough and Biscay Bay mostly in depths over 2000 m (Oliver et al. 2009); Brazil–Campos Basin, 1200–1972 m (present study).

#### Material examined.

MNRJ 19114 (# 68, 2003), 4 valves; MZUSP 99977 (# 68, 2003), 4 valves; IBUFRJ 16161 (# 68, 2002), 3 valves; IBUFRJ 19001 (# 10, 2002), 5 valves; IBUFRJ 19002 (# 42, 2002), 3 valves; IBUFRJ 19003 (# 47, 2002), 1 valves; IBUFRJ 19004 (# 48, 2002), 7 valves; IBUFRJ 19005 (# 52, 2002), 1 valve; IBUFRJ 19006 (# 58, 2002), 6 valves; IBUFRJ 19007 (# 62, 2002), 3 valves; IBUFRJ 19008 (# 63, 2002), 6 valves; IBUFRJ 19009 (# 73, 2002), 14 valves; IBUFRJ 19010 (# 77, 2002), 2 valves; IBUFRJ 19011 (# 78, 2002), 4 valves; IBUFRJ 19012 (# 83, 2002), 2 valves; IBUFRJ 19013 (# 87, 2002), 6 valves; IBUFRJ 19014 (# 46, 2003), 1 valve; IBUFRJ 19015 (# 48, 2003), 15 valves; IBUFRJ 19016 (# 52, 2003), 1 valve; IBUFRJ 19017 (# 53, 2003), 16 valves; IBUFRJ 19018 (# 58, 2003), 3 valves; IBUFRJ 19019 (# 61, 2003), 2 valves; IBUFRJ 19020 (# 63, 2003), 16 valves; IBUFRJ 19021 (# 68, 2003), 14 valves; IBUFRJ 19022 (# 72, 2003), 1 valve; IBUFRJ 19023 (# 73, 2003), 8 valves; IBUFRJ 19024 (# 78, 2003), 14 valves; IBUFRJ 19025 (# 82, 2003), 1 valve; IBUFRJ 19026 (# 86, 2003), 4 valves.

#### Characterization.

Shell H/L ratio about 1.04. W/H ratio approximately 0.86. Muscle scars rarely visible; anterior adductor scar 2/3 of posterior scar. Anterior and posterior parts of the hinge plate usually of same length. Width of posterior row of teeth occupies about 65% of width of hinge plate, which is thin for its size (whp/H approximately 0.07). Posterior part of the hinge plate longer than anterior one. Prodissoconch smooth, with length approximately 120 µm.

#### Remarks. 

This species was recently well described and for this reason we add only new information on the proportions of the shell (H/L and width) and hinge plate characteristics. Sanders and Allen (1973) noted no evident muscle scars or pallial line. In the Campos Basin specimens, no pallial line is discernible on the valves, but faint muscle scars are apparent in some specimens. It is a common species in the Atlantic, and its occurrence in the Campos Basin was expected, since it was previously recorded from the northern Brazilian coast (Pernambuco) and from Argentina. The present study provides new points to the geographical distribution of *Pristigloma alba*, and is also the shallowest (1200 m) record for the species, which was previously known only from abyssal depths (2100–4898 m).

### Microgloma mirmidina

(Dautzenberg & Fischer, 1897)

http://species-id.net/wiki/Microgloma_mirmidina

Figs 9–13

Leda mirmidina Dautzenberg & Fischer, 1897: 208, pl. 6, figs 11–14; Dautzenberg 1927: 292, pl. 8, figs 27–30.
Nuculana mirmidina : Clarke 1962: 53.
Microgloma mirmidina : La Perna 2008: 154, 155, fig 6.

#### Geographical distribution. 

Southeast of Flores, Azores, 1846 m (La Perna 2008). Campos Basin 1050–1950 m (present study).

#### Material examined. 

MNRJ 19115 (#68, 2003), 6 valves; MZSP 99978 (#71, 2003), 7 valves; IBUFRJ 15889 (#67, 2003), 5 valves; IBUFRJ 17501 (#87, 2003), 4 valves; IBUFRJ 19084 (# 10, 2001), 4 valves; IBUFRJ 19085 (# 28, 2001), 1 valve; IBUFRJ 19086 (# 42, 2002), 1 valve; IBUFRJ 19087 (# 48, 2002), 6 valves and 2 specimens; IBUFRJ 19088 (# 51, 2002), 1 valve; IBUFRJ 19089 (# 53, 2002), 6 valves and 1 specimen; IBUFRJ 19090 (# 57, 2002), 1 valve IBUFRJ 19091 (# 62, 2002), 4 valves; IBUFRJ 19092 (# 63, 2002), 10 valves; IBUFRJ 19093 (# 68, 2002), 1 valve; IBUFRJ 19094 (# 73, 2002), 6 valves and 1 specimen; IBUFRJ 19095 (# 75, 2002), 1 valve; IBUFRJ 19096 (# 77, 2002), 12 valves and 1 specimen; IBUFRJ 19097 (# 78, 2002), 4 valves; IBUFRJ 19098 (# 81, 2002), 2 valves; IBUFRJ 19098 (# 81, 2002), 2 valves; IBUFRJ 19099 (# 83, 2002), 4 valves and 1 specimen; IBUFRJ 19100 (# 85, 2002), 1 valve; IBUFRJ 19101 (# 86, 2002), 6 valves; IBUFRJ 19102 (# 87, 2002), 5 valves; IBUFRJ 19103 (# 50A, 2003), 1 valve; IBUFRJ 19105 (# 61, 2003), 3 valves; IBUFRJ 19106 (# 63, 2003), 2 valves; IBUFRJ 19107 (# 72, 2003), 2 valves and 1 specimen; IBUFRJ 19108 (# 73, 2003), 4 valves; IBUFRJ 19109 (# 77, 2003), 5 valves and 3 specimens; IBUFRJ 19110 (# 78, 2003), 4 valves; IBUFRJ 19111 (# 82, 2003), 2 valves; IBUFRJ 19112 (# 84, 2003), 2 valves; IBUFRJ 19113 (# 86, 2003), 2 valves; IBUFRJ 19114 (# C10, 2008), 1 valve.

**Figure:**
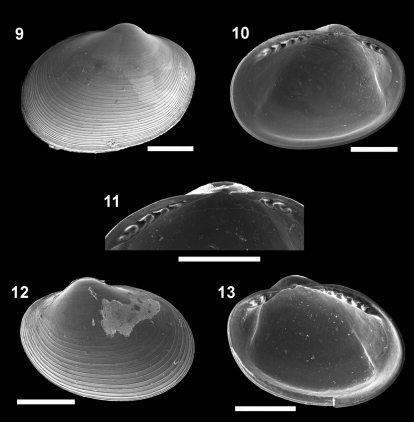
**Figures 9–13.** *Microgloma mirmidina* (Dautzenberg & Fischer, 1897). External view, left valve **9** internal view, right valve **10** Detail of the hinge plate **11** External view, right valve **12** internal view, left valve **13** All from the lot IBUFRJ 15889. Scale bars A= 200 μm; B, E = 300 μm; C = 100 μm; D = 250μm.

#### Characterization.

 Shell H/L approximately 0.79 (n 10, min 0.75, max 0.83), W/H approx. 0.59(n 10, min 0.53, max 0.63). Posterior part of the hinge plate shorter than anterior one. Width of both the anterior and posterior rows of teeth occupies nearly half of the width of the hinge plate, which is moderately thick for its size (whp/H about 0.11). Prodissoconch smooth, length approximately 176 µm.

#### Remarks. 

The radial sculpture on the prodissoconch surface is absent in *Microgloma mirmidina*, but admittedly it is not always clearly developed in the other species of *Microgloma* (La Perna 2008). *Microgloma mirmidina* differs from other species of the genus in the elongated outline, more inflated shells and small hinge plate. In relation to the type material of *Microgloma mirmidina* figured by La Perna (2008) the Campos Basin material presents a smaller hinge plate. The figured specimens presents H/L ratio of 0.72, 0.76 and 0.83 (La Perna 2008 fig 6 B, E, I, respectively). Despite geographical distance between the two records of the present species, these conchological differences are not pronounced enough to affirm they belong to distinct species.

### Microgloma macaron sp. n.

urn:lsid:zoobank.org:act:31275EB9-546D-414B-82B0-C59CA55F8C93

http://species-id.net/wiki/Microgloma_macaron

Figs 14 22

#### Holotype. 

**Figures 14–18.**
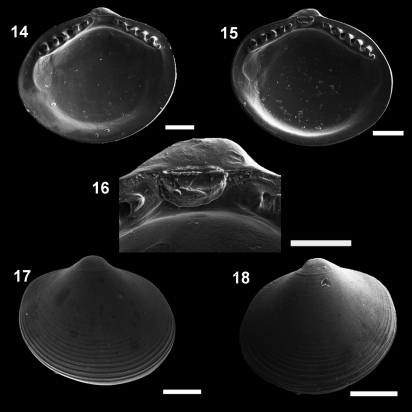
*Microgloma macaron* sp. n. Internal view, right valve **14** left valve **15** detail of the hinge plate and ligament **16** External view, right valve **17** left valve **18** Holotype MNRJ 19112 (14,16,18). Paratype IBUFRJ 15297 **15, 17** Scale bars: 14, 16= 200 µm; 15, 18 = 300 µm; 17 = 250 µm.

MNRJ 19.112 (Figs 14, 16, 18).

#### Type locality. 

Campos Basin, #54, 12/12/2002, 750m, 21°57'17,5"S, 39°56' 01,1"W.

**Paratypes.** IBUFRJ 15297, 8 valves and 2 specimens; MZUSP 99979 , 4 valves; USNM 1156943 , 6 valves; MNHN 24596, 6 valves; all from the type locality.

#### Etymology. 

The species epithet refers to the French macaroon cookie (“macaron" in the French language), which has a similar appearance to the articulated valves. The species epithet is proposed as a noun in apposition.

#### Material examined.

IBUFRJ 19145 (# 32, 2002), 3 valves; IBUFRJ 19146 (# 33, 2002), 1 valve; IBUFRJ 19147 (# 34, 2002), 1 valve; IBUFRJ 19148 (# 36, 2002), 1 valve; IBUFRJ 15482 (# 54, 2002), 6 valves; IBUFRJ 17033 (# 59, 2002), 1 specimen; IBUFRJ 19150 (# 61, 2002), 3 specimens; IBUFRJ 16074 (# 64, 2002), 14 valves and 1 specimen; IBUFRJ 15141 (# 69, 2002), 7 valves; IBUFRJ 15285 (# 74, 2002), 2 valves; IBUFRJ 15635 (# 54, 2003), 32 valves and 5 specimens; IBUFRJ 19152 (# 59, 2003), 6 valves and 1 specimen; IBUFRJ 19153 (# 61, 2003), 1 valve; IBUFRJ 19153 (# 61, 2003), 1 valve; IBUFRJ 19154 (# 64, 2003), 18 valves and 4 specimens; IBUFRJ 19155 (# 69, 2003), 4 valves; IBUFRJ 19156 (# D11, 2008), 1 specimen; IBUFRJ 19157 (# G12, 2008), 1 specimen; IBUFRJ 19158 (# A7, 2008), 10 specimens; IBUFRJ 19159 (# A7, 2008), 1 specimen; IBUFRJ 19160 (# A7, 2008), 2 valves and 5 specimens; IBUFRJ 19161 (# D7, 2008), 1 specimen; IBUFRJ 19162 (# D7, 2008), 6 specimens; IBUFRJ 19163 (# CANAC7, 2008), 1 specimen; IBUFRJ 19164 (# H7, 2008), 1 specimen; IBUFRJ 19165 (# H7, 2008), 1 specimen; IBUFRJ 19166 (# H7, 2008), 5 specimens; IBUFRJ 19167 (# I7, 2008), 2 specimens; IBUFRJ 19168 (# A7, 2009), 3 specimens; IBUFRJ 19169 (# A7, 2009), 5 specimens; IBUFRJ 19170 (# D6, 2009), 1 specimen; IBUFRJ 19171 (# D7, 2009), 1 specimen; IBUFRJ 19172 (# H7, 2009), 12 specimens; IBUFRJ 19173 (# CANAC7, 2009), 1 specimen; IBUFRJ 19174 (# CANAC7, 2009), 1 specimen; IBUFRJ 19175 (# CANG7, 2009), 3 specimens; IBUFRJ 19177 (# 64, 2003), 4 specimens.

**Figures 19–22.**
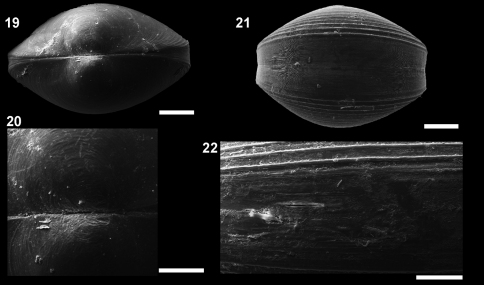
*Microgloma macaron* sp. n. Dorsal view **19** prodissoconch **20** (IBUFRJ 15297). Ventral margin view, extended margin **21** periostracum of the ventral margin **22** (IBUFRJ 19177). Scale bars: **19, 21** = 200 µm; **20, 22** = 100 µm.

#### Diagnosis.

Shell minute, ovate. Entire shell margin thickened and covered by an extension of the periostracum. Prodissoconch surface with one subtle radial striae.

#### Description.

Shell minute, ovate, H/L ratio about 0.81 (n 38, min 0.75, max 0.92), W/H ratio about 0.84 (n 14, min 0.77, max 0.87), glossy, translucent, robust for its size, equilateral; umbones prominent, large, posterior to midline, orthogyrous. Antero–dorsal margin straight, oblique; anterior margin rounded, extended. Antero–ventral margin, postero–ventral margin shorter and rising up to the short posterior end; posterior margin slightly truncated, forming a small shoulder. Entire shell margin thickened and covered by an extension of the periostracum, resembling a macaroon in ventral view (Figs 21–22). Surface with commarginal growth lines. Hinge plate with 5–7 anterior and 4–5 posterior teeth, interrupted by a large, rectangular and shallow resilifer. Width of both anterior and posterior row of teeth occupies about 70% of width of hinge plate, which is thick for its size (whp/H approximately Campos Basin, #54, 12/12/2002 0.14). Posterior part of the hinge plate shorter than anterior one. Prodissoconch surface nacreous, with one subtle radial striae (Fig 20), and length approximately 244 µm. Maximum adult shell length 1.20 mm.

**Table 3.** Measurements of the type material. * Width = width of a single valve.

***Microgloma macaron* sp. n.**			
	Length	Height	Width*
MNRJ 19.112	1.20	1.00	0.36
IBUFRJ 15.297	1.11	0.87	0,31
IBUFRJ 15.297	1.11	0.89	0.36
IBUFRJ 15.297	1.11	0.91	0.36
IBUFRJ 15.297	1.11	0.84	0.33
IBUFRJ 15.297	1.11	0.89	0.36
IBUFRJ 15.297	1.11	0.89	0.36
IBUFRJ 15.297	1.16	0.93	0.38
IBUFRJ 15.297	1.11	0.91	0.33
IBUFRJ 15.297	1.13	0.82	0.31
IBUFRJ 15.297	1.13	0.89	0.33
MZSP 99.979	1.11	0.87	0.33
MZSP 99.979	1.13	1.04	0.36
MZSP 99.979	1.16	0.93	0.38
MZSP 99.979	1.11	0.91	0.31
USNM 1156943	1.11	0.89	0.36
USNM 1156943	1.11	0.89	0.36
USNM 1156943	1.13	0.91	0.36
USNM 1156943	1.13	0.89	0.38
USNM 1156943	1.13	0.91	0.33
USNM 1156943	1.11	0.89	0.36
MNHN 24596	1.11	0.91	0.33
MNHN 24596	1.18	0.93	0.38
MNHN 24596	1.11	0.91	0.36
MNHN 24596	1.11	0.89	0.38
MNHN 24596	1.11	0.91	0.36
MNHN 24596	1.04	0.87	0.38

#### Remarks. 

In some other species of *Microgloma*, the shell is expanded ventrally, around the valve, which enlarges the internal volume of the animal and counterbalances the effects of miniaturization (Ockelmann and Warén 1998, La Perna 2008). However, in *Microgloma macaron* this expansion is thicker, and the periostracum surrounds the entire margin (Fig. 22). This characteristic of the periostracum deserves special attention since it not only covers the shell to the margins in the usual way, but is more conspicuous in this area, giving the impression that valves do not articulate. We are not sure how this system works, and how the animal, in spite of having these fibers surrounding the valve apertures, can have water flux in the mantle cavity.

Compared to *Microgloma pusilla* and *Microgloma mirmidina*, *Microgloma macaron* is distinguished by the ovate outline, umbones at midline and much more projecting, and a thicker hinge plate. *Microgloma macaron* is similar to *Microgloma yongei* in outline, but compared with the paratypes figured by Ockelmann and Warén (1998, page 14, fig 6–D), the former has more prominent umbones, a thicker hinge plate, as well as a larger resilifer. *Microgloma macaron* can be distinguished from *Microgloma tumidula* by the shape of the teeth, which are not as inclined as in this latter species. The anterior and posterior areas of the hinge plate form a less obtuse angle compared to those of *Microgloma tumidula*.

Except for two, probably worn, valves found at twostations at approx. 3000 m depth, and one at 1970 m, this species is concentrated at depths between 400–750 m. Untill now this species is reccorded solely in Campos Basin.

### Microgloma nhanduti sp. n.

urn:lsid:zoobank.org:act:511F7840-3490-4EA1-A48F-CB56C60329FC

http://species-id.net/wiki/Microgloma_nhanduti

Figs 23 31

#### Holotype.

 MNRJ 19.113 (Figs 26, 28).

#### Type locality.

 Campos Basin, #54, 12/12/2002, 750m, 21°57'17,5"S, 39°56'01,1"W.

#### Paratypes.

IBUFRJ 14991 (# 54, 2002), 2 valves and 1 specimen; IBUFRJ 19176 (# 64, 2003), 1 specimen; MZSP 99980 (# 54, 2002), 5 valves; USNM 1156944 (# 54, 2002), 5 valves; MNHN 24597 (# 54, 2002), 2 valves 1 specimen.

#### Etymology.

Nhanduti is a Tupi–Guarani term (the language spoken by the largest groups of native people living in Brazil prior to the European colonization) for a spider web–like structure, similar to those present on the prodissoconch of this species. The species epithet is proposed as a noun in apposition.

#### Material examined. 

IBUFRJ 15140 (# 69, 2002), 17 valves and 2 specimens; IBUFRJ 15283 (# 69, 2002), 6 valves; IBUFRJ 19115 (# 33, 2002), 1 valve; IBUFRJ 19116 (# 64, 2002), 16 valves and 1 specimen; IBUFRJ 19117, (# 49, 2003), 7 valves; IBUFRJ 19118, (# 54, 2003), 1 valve; IBUFRJ 19119, (# 59, 2003), 10 valves and 2 specimens; IBUFRJ 19121, (# 34, 2002), 1 valve; IBUFRJ 19122, (# 37, 2002), 1 valve; IBUFRJ 19123, (# 38, 2002), 1 valve; IBUFRJ 19124, (# 64, 2003), 12 valves and 1 specimen; IBUFRJ 19125, (# 75, 2003), 2 valves; IBUFRJ 19126, (# A7, 2008), 4 specimens; IBUFRJ 19127, (# A7, 2008), 1 valve; IBUFRJ 19128, (# A7, 2008), 1 valve and 3 specimens; IBUFRJ 19129, (# D7, 2008), 1 specimen; IBUFRJ 19130 (# H7, 2008), 2 specimens; IBUFRJ 19131 (# D6, 2008), 3 specimens; IBUFRJ 19132 (# H7, 2008), 1 specimen; IBUFRJ 19133 (# H7, 2008), 4 valves; IBUFRJ 19134 (# D6, 2008), 1 specimen; IBUFRJ 19135 (# I7, 2008), 1 specimen; IBUFRJ 19136 (# A7, 2009), 2 specimens; IBUFRJ 19137 (# A7, 2009), 6 specimens; IBUFRJ 19138 (# D6, 2009), 4 specimens; IBUFRJ 19139 (# H7, 2009), 10 specimens; IBUFRJ 19140 (# CANAC7, 2009), 5 specimens and 2 valves; IBUFRJ 19141(# CANAC7, 2008), 1 specimen; IBUFRJ 19142 (# I7, 2008), 2 specimens; IBUFRJ 19143 (# CANAC7, 2008), 6 specimens; IBUFRJ 19144 (# D7, 2008), 1 specimen.

**Figure:**
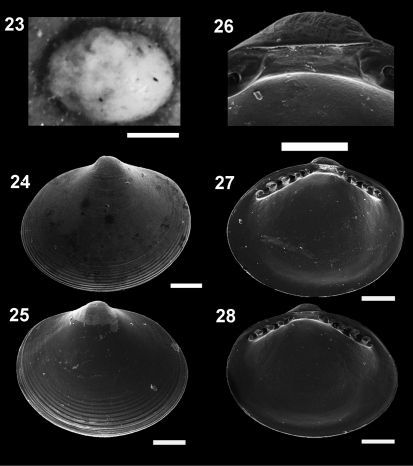
**Figures 23–28.** *Microgloma pusilla* (Jeffreys, 1879) USNM 199712 **23** *Microgloma nhanduti* sp. n. External view, leftt valve **24** IBUFRJ 15283, right valve **25** IBUFRJ 14991. Internal view holotype MNRJ 19.113, detail of the reslifer and umbo **26** right valve **27** left valve **28** Scale bars: 23 = 500 µm; 26 = 100 µm; 24–25, 27–28 = 200 µm.

**Figures 29–31.**
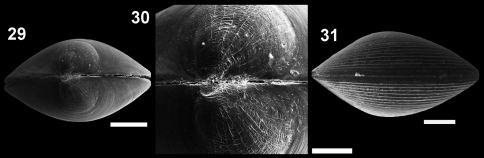
*Microgloma nhanduti* sp. n. Dorsal view IBUFRJ 19131 **29** prodissoconch surface **30** ventral margin IBUFRJ 19176 **31** Scale bars: 29, 30 = 200 µm, 31 = 80 µm.

#### Diagnosis.

Shell minute, ovate. Entire margin slightly thickened. Hinge plate moderately thick. Prodissoconch with web–like striae.

#### Description. 

Shell minute, ovate, H/L ratio about 0.78 (n 29, min 0.74, max 0.81), Width of both valves/H ratio about 0.7 (n 11, min 0.67, max 0.77), inequilateral, translucent, glossy; umbones moderately prominent, large, posterior to midline, orthogyrous. Antero–dorsal margin convex and oblique; anterior margin rounded and projected. Antero–ventral margin convex, postero–ventral margin shorter and rising up to short and rounded posterior end; postero–dorsal margin convex, but forming a small shoulder. Entire shell margin slightly thickened. Surface with subtle commarginal growth lines. Hinge plate with 5–7 anterior and 4–5 posterior teeth, interrupted by a shallow rectangular hinge plate. Width of both anterior and posterior row of teeth occupies about 70% of width of hinge plate, which is thick for its size (whp/H approximately 0.14). Posterior part of the hinge plate shorter than anterior one. Prodissoconch surface nacreous, with several radial striae, resembling a spider’s web. Prodissoconch length approximately 246 µm.Maximum adult shell length 1.16 mm.

**Table 4.** Measurements of the type material. * Width = width of a single valve.

***Microgloma nhanduti* sp. n.**			
	Length	Height	Width*
MNRJ 19.113	1.12	0.90	0.29
IBUFRJ 14.991	1.11	0.84	0.24
IBUFRJ 14.991	1.04	0.82	0.29
IBUFRJ 14.991	1.09	0.89	0.29
IBUFRJ 19.176	1.13	0.89	0.29
MZSP 99.980	1.09	0.82	0.29
MZSP 99.980	1.09	0.82	0.27
MZSP 99.980	0.98	0.76	0.24
MZSP 99.980	1.09	0.82	0.27
MZSP 99.980	1.11	0.82	0.27
MZSP 99.980	1.11	0.84	0.22
USNM 1156944	1.11	0.87	0.27
USNM 1156944	1.11	0.87	0.29
USNM 1156944	0.96	0.78	0.27
USNM 1156944	1.07	0.80	0.24
USNM 1156944	1.11	0.89	0.27
MNHN 24596	1.07	0.87	0.22
MNHN 24596	1.07	0.87	0.22
MNHN 24596	1.09	0.80	0.22

#### Remarks.

*Microgloma nhanduti* sp. n. is similar to *Microgloma pusilla* and *Microgloma tumidula* in the oval outline. but has more prominent umbones (Figs 26–28). Compared to *Microgloma macaron* sp. n. and *Microgloma yongei*, *Microgloma nhanduti* sp. n. has a more elongated outline, a longer anterior area, and a more evident prodissoconch sculpture, with a web–like pattern (Figs 29–30). In *Microgloma nhanduti* sp. n. the umbo is not as prominent and the shell margin not as thick as in *Microgloma macaron* sp. n.Untill now this species is reccorded solely in Campos Basin.

## Discussion

Ockelmann and Warén (1998) carefully evaluated the systematic position of the Nuculoidea and Nuculanoidea, and placed *Microgloma* within the Nuculanidae (a position with which we agree). However, these authors did not use the subfamily rank introduced by Allen and Hannah (1986). Considering differences such as prominence of the rostral area, presence of carena and keels, foot grooves, and the characteristics of the ligament among some groups within the Nuculanidae such as in *Ledella* Verrill and Bush, 1897, *Propeleda* Iredale, 1924, and *Nuculana* Link, 1807, we believe that the proposed subfamilies should be used for taxonomic purposes. Whether they represent a natural division or not is a matter to be discussed later in a phylogenetic study.

The genus *Microgloma* is still in need of review since, as stated by Ockelmann and Warén (1998), “similarity in hinge structure to juvenile specimens of *Yoldiella* and other nuculanids directly suggests progenesis. We assume that the species of *Microgloma* simply are species derived from *Yoldiella* or *Ledella*, which mature at a much smaller size than normal in these taxa. (…) Possibly the genus *Microgloma* is polyphyletic, since progeneses may have taken place more than once. This will be difficult to prove or disprove." The similarities with some species of the genera cited above may confuse many researches in the identification of *Microgloma* species and the validity or status of the genus must be revisited. We believe this is an issue to be resolved with molecular analysis. At present we can only assume the genus to be valid.

The sculpture pattern on the prodissoconch surface is not a character commonly used in taxonomy of the protobranchs and, considering the confused taxonomy of the protobranchs (as seen by the genus *Microgloma*), we believe it might be useful to better determine the genera. This character has been recorded recently in the literature, and some species, from different families, show particular patterns. The reticulated sculpture on the prodissoconch surface seems to be a common character for the Nuculanidae, and has been recorded for the genera *Nuculana*, *Sacella* Woodring 1925 (Allen 1993, Ockelmann and Warén 1998, La Perna 2007), *Propeleda* (seen by Natalia Benaim; unpublished data), and the Bathyspinulidae, in *Tindariopsis agathida* Dall, 1889 (seen by the present authors; unpublished data). Some members of Nuculidae have ridges or knobs on the prodissoconch surface (Gofas and Salas 1996, Zardus 2002). The genus *Yoldiella* presents a smooth prodissoconch surface, but the species *Yoldiella philippiana* (Nyst, 1845) presents a radial prodissoconch sculpture with a web-like pattern (Ockelmann and Warén 1998, Salas 1996) as seen here in *Microgloma nhanduti* sp. n. Salas (1996) also illustrated radial ridges on the prodissoconch of *Microgloma pusilla* and *Microgloma tumidula* from the Iberian Peninsula. The radial ridges present in *Microgloma macaron* sp. n. and *Microgloma nhanduti* sp. n. are distinctive characters that should be considered in future descriptions of species of *Microgloma* to aid in resolving the status of the genus.

## Conclusion

The apparent absence of species of the genus *Microgloma* along the Brazilian coast wais an artifact, reflecting the logistical difficulties in obtaining material from these depths. Once this material became available, additions to the fauna was brought to light. The description of two new species of *Microgloma* and the new information on the conchology, and bathymetric and geographical distributions of *Microgloma mirmidina* and *Pristigloma alba* contribute to knowledge of the biodiversity of deep–sea mollusks of the Campos Basin and Brazil.

## Supplementary Material

XML Treatment for Pristigloma alba

XML Treatment for Microgloma mirmidina

XML Treatment for Microgloma macaron

XML Treatment for Microgloma nhanduti
